# Frequência Cardíaca de Repouso para Avaliar Pacientes com Insuficiência Cardíaca: É Tudo o que Precisamos

**DOI:** 10.36660/abc.20240521

**Published:** 2024-09-06

**Authors:** Humberto Villacorta

**Affiliations:** 1 Universidade Federal Fluminense Niterói RJ Brasil Universidade Federal Fluminense, Niterói, RJ – Brasil

**Keywords:** Frequência Cardíaca, Insuficiência Cardíaca, Eletrocardiografia

A frequência cardíaca (FC) é um importante marcador de prognóstico em doenças cardiovasculares.^1^ Está presente como um preditor de sobrevivência na própria natureza. Por exemplo, animais com baixa FC vivem muito mais do que animais com alta FC.^1^ Na população geral, a FC tem sido relacionada à mortalidade, conforme demonstrado no Estudo de Framingham.^2^ Além disso, desde a década de 1980, sabe-se que a FC de repouso é um fator prognóstico em pacientes com doença arterial coronariana.^3,4^ Dados do Coronary Artery Surgery Study (CASS) mostraram que ele prediz morbidade (taxa de readmissão hospitalar), bem como mortalidade total e cardiovascular.^4^

**Figura 1:**
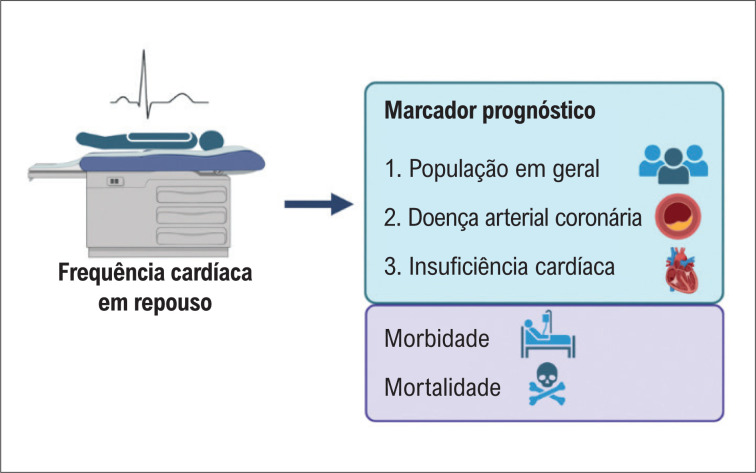
Demonstrou-se que a frequência cardíaca em repouso prediz morbidade e mortalidade em muitas condições cardiovasculares e até mesmo na população em geral.

Na insuficiência cardíaca (IC) a FC de repouso também é um marcador prognóstico, conforme mostrado na Figura 1.^5^ O tratamento da IC com fração de ejeção reduzida inclui a utilização de betabloqueadores.^6–8^ Embora os betabloqueadores tenham muitos mecanismos através dos quais podem beneficiar os pacientes com IC, a redução da FC provavelmente contribui para os efeitos benéficos desta classe. Entretanto, mesmo em doses máximas toleradas de betabloqueadores, alguns pacientes podem permanecer com FC >70 bpm (a faixa recomendada para pacientes com IC é de 50-60 bpm).^9^ Por esta razão, foi desenvolvida uma nova classe de medicamentos. A ivabradina é um redutor seletivo da FC que atua inibindo os canais *if* do nó sinusal.^9^ A ivabradina foi testada contra um placebo no estudo SHIFT, em pacientes com IC sintomática, ritmo sinusal, fração de ejeção do ventrículo esquerdo ≤35% e FC >70 bpm apesar do tratamento otimizado para IC.^9^ A ivabradina reduziu o *endpoint* composto de mortalidade cardiovascular ou hospitalização por IC.^9^ Em uma subanálise, observou-se que a magnitude da redução da FC pelo betabloqueador mais ivabradina, em vez da dose de base do betabloqueador, determinou principalmente o efeito subsequente nos resultados.^10^

Assim, a FC é um marcador importante na avaliação de pacientes com IC, e as decisões sobre introdução, ajuste de dose e retirada de alguns medicamentos são baseadas neste parâmetro. No entanto, a comunidade médica sempre se questionou se o monitoramento ambulatorial da FC utilizando um sistema Holter de 24 horas forneceria informações diferentes da FC em repouso. Neste número dos Arquivos Brasileiros de Cardiologia, o estudo de Camazzola et al. comparou a FC de repouso com a observada pelo sistema Holter de 24 horas em pacientes com IC com fração de ejeção reduzida e ritmo sinusal.^11^ Os autores concluem que a FC obtida no eletrocardiograma (ECG) de repouso apresentou excelente correlação com a FC obtida no Holter de 24 horas, exceto naqueles com FC <70 bpm no ECG. Os autores afirmam que neste último grupo deve ser considerado o Holter de 24 horas.

O estudo é original e possui uma boa metodologia e parabenizamos os autores por isso. Tem o mérito de garantir que a FC de repouso não é muito diferente daquela observada no acompanhamento ambulatorial de pacientes com IC. Porém, do ponto de vista prático, este estudo não altera a nossa prática, uma vez que todos os dados que temos com betabloqueadores e ivabradina provêm da avaliação da FC de repouso. Portanto, de acordo com as diretrizes da IC, as decisões devem ser tomadas tendo como referência a FC de repouso.^12^ Os autores sugerem que em pacientes com FC<70 bpm o Holter de 24 horas deve ser considerado, mas o estudo carece de informações para essa recomendação, uma vez que foi transversal e não foram medidos eventos. A FC de repouso entre 50-60 bpm foi na verdade a meta no estudo SHIFT e nenhum procedimento adicional foi realizado no estudo SHIFT quando essa faixa de FC foi atingida.^9^

Em resumo, parabenizamos os autores por este elegante estudo. Do ponto de vista mecanicista, agrega informações ao nosso conhecimento na área. Contudo, até que novos dados longitudinais de estudos multicêntricos sejam publicados, as decisões devem ser tomadas com base na FC de repouso.
