# Supplementary material for: Frequência Cardíaca de Repouso para Avaliar Pacientes com Insuficiência Cardíaca: É Tudo o que Precisamos
Source: Arq Bras Cardiol. 2024 Sep 6;121(8):e20240521. [Article in Portuguese] doi: 10.36660/abc.20240521 (PMC11495799; doi:10.36660/abc.20240521)
Supplement: Translation

# Translation: Resting Heart Rate to Assess Patients with Heart Failure: That is All We Need

**DOI:** 10.36660/abc.20240521i

**Authors:** Humberto Villacorta

**Affiliations:** 1 Niterói RJ Brazil Universidade Federal Fluminense, Niterói, RJ – Brazil

**Keywords:** Heart Rate, Heart Failure, Electrocardiography

Heart rate (HR) is an important marker of prognosis in cardiovascular diseases.^1^ It is present as a predictor of survival in nature itself. For example, animals with a low HR live much longer than animals with a high HR.^1^ In the general population, HR has been related to mortality, as demonstrated in the Framingham Study.^2^ Additionally, since the 1980s, it has been known that resting HR is a prognostic factor in patients with coronary artery disease.^3,4^ Data from the Coronary Artery Surgery Study (CASS) showed that it predicts morbidity (hospital readmission rate) as well as total and cardiovascular mortality.^4^

In heart failure (HF) resting HR is a prognostic marker as well, as shown in Figure 1.^5^ The treatment of HF with reduced ejection fraction includes the utilization of beta-blockers.^6–8^ Although beta-blockers have many mechanisms through which they may benefit patients with HF, HR reduction probably contributes to the beneficial effects of this class. However, even on maximum tolerated doses of beta-blockers, some patients may remain with HR >70 bpm (recommended range for patients with HF is 50-60 bpm).^9^ For this reason, a new class of drugs was developed. Ivabradine is a selective HR reductor that works by inhibiting the *if* channels in the sinus node.^9^ Ivabradine was tested against a placebo in the SHIFT study, in patients with symptomatic HF, sinus rhythm, left ventricular ejection fraction ≤35%, and HR ≥70 bpm despite optimized HF treatment.^9^ Ivabradine reduced the composite endpoint of cardiovascular mortality or HF hospitalization.^9^ In a subanalysis, it was observed that the magnitude of HR reduction by beta-blocker plus ivabradine, rather than background beta-blocker dose, primarily determined subsequent effect on outcomes.^10^

**Figure 1:** Resting heart rate has been shown to predict morbidity and mortality in many cardiovascular conditions and even in the general population.

Thus, HR is an important marker in the evaluation of patients with HF, and decisions on the introduction, dose adjustments, and withdrawal of some drugs are based on this parameter. Nevertheless, the medical community has always wondered whether ambulatorial monitoring of HR employing a 24-hour Holter system would provide different information than resting HR. In this issue of *Arquivos Brasileiros de Cardiologia*, a study by Camazzola et al. compared resting HR with that observed with the 24-hour Holter system in patients with HF with reduced ejection fraction and sinus rhythm.^11^ The authors conclude that HR obtained from the resting electrocardiogram (ECG) had an excellent correlation with the HR obtained from the 24-hour Holter, except in those with HR <70 bpm on the ECG. The authors state that in the latter group, 24-hour Holter should be considered.

The study is original and has a good methodology and we congratulate the authors for that. It has the merit of reassuring that resting HR is not very different from that observed in the ambulatory monitoring of patients with HF. However, from a practical standpoint, this study does not change our practice, since all data that we have with beta-blockers and ivabradine come from assessment of resting HR. Therefore, according to HF guidelines, the decisions must be made using resting HR as a reference.^12^ The authors suggest that in patients with HR<70 bpm 24-hour Holter should be considered but the study lacks information for that recommendation since it was cross-sectional and no events were measured. Resting HR between 50-60 bpm was actually the target in the SHIFT study and no additional procedures were done in the SHIFT study when this range of HR was achieved.^9^

In summary, we congratulate the authors for this elegant study. From a mechanistic point of view, it adds information to our knowledge in the field. Nevertheless, until new longitudinal data from multicentric studies are published, decisions should be made based on resting HR.
